# Serum CEA and CA 15-3 as prognostic factors in primary breast cancer

**DOI:** 10.1038/sj.bjc.6600248

**Published:** 2002-04-22

**Authors:** F G Ebeling, P Stieber, M Untch, D Nagel, G E Konecny, U M Schmitt, A Fateh-Moghadam, D Seidel

**Affiliations:** Institute of Clinical Chemistry, Klinikum Grosshadern, Ludwig-Maximilians-University Munich, Germany; Department of Obstetrics and Gynecology, Klinikum Grosshadern, Ludwig-Maximilians-University Munich, Germany

**Keywords:** primary breast cancer, tumour markers, CEA, CA 15-3, prognosis, multivariate analysis

## Abstract

In the present study, we investigated the association of the serum levels of the tumour markers carcinoembryonic antigen and cancer antigen 15-3 with disease free survival and death from disease in 1046 women with breast cancer without metastases at the time of primary diagnosis in relation to age and the established prognostic factors tumour size, lymph node status, histological grading and hormone receptor status. We found that elevated pre-operative serum marker values were correlated with early relapse (cancer antigen 15-3; *P*=0.0003) and death from disease (carcinoembryonic antigen, cancer antigen 15-3; *P*=0.0001 both) in univariate analyses. By comparing pre- and post-operative values we found a decline in values post-surgery. In those patients where marker levels of carcinoembryonic antigen decreased more than 33%, a significantly higher risk for relapse and death from disease (both *P*=0.0001) in univariate analyses was observed. In multivariate analysis this decrease of carcinoembryonic antigen proved to be an independent prognostic factor. The results for cancer antigen 15-3 were comparable to carcinoembryonic antigen in univariate analyses but showed no significance in multivariate analysis. In this study the post-operative decrease of the serum tumour marker carcinoembryonic antigen was a strong independent prognostic factor for disease free survival and death from disease in breast cancer patients.

*British Journal of Cancer* (2002) **86**, 1217–1222. DOI: 10.1038/sj/bjc/6600248 www.bjcancer.com

© 2002 Cancer Research UK

## 

As adjuvant therapy for breast cancer has become more generally used, there seems to be a decreased need for prognostic factors. Nonetheless it remains a challenge to predict which patients are at greatest risk of relapse and thus may benefit most from adjuvant therapy (McGuire and Clark, 1992; Mansour *et al*, 1994). In addition to traditional prognostic factors in breast cancer such as tumour size, axillary lymph node status, histological grading and hormone receptor status (Clark *et al*, 1987; Chevallier *et al*, 1988; Carter *et al*, 1989; Nomura *et al*, 1992; Carriaga and Henson, 1995), newer parameters like Her-2/neu, cathepsin D or urokinase plasminogen activator (uPA) and plasminogen activator inhibitor 1 (PAI-1) are under consideration (Mansour *et al*, 1994; Schmitt *et al*, 1997). Circulating tumour markers as Carcinoembryonic Antigen (CEA) and Cancer Antigen 15-3 (CA 15-3) have become well established diagnostic tools as fast, non-invasive, reproducible and quantitative parameters in follow-up care and monitoring therapy of breast cancer patients. However, the potential role of these factors in prognosis has only been studied in a few investigations, which came to inconsistent conclusions (Tondini *et al*, 1989; Lamerz *et al*, 1993; Shering *et al*, 1998). The only multivariate analysis based on a large number of patients (Shering *et al*, 1998) investigated CA 15-3 alone.

In the present study we analysed the serum markers CEA and CA 15-3 at the time of primary intervention, and related levels of both markers to patient outcome using both univariate and multivariate analysis.

## MATERIALS AND METHODS

### Patients

All female primary breast cancer patients, who underwent surgery between September 1985 and June 1998 at the University Department of Obstetrics and Gynecology, Klinikum Grosshadern, Munich, were considered for inclusion in this retrospective study. Patients were excluded if any other malignancy was known from their past history or if staging investigations at the time of diagnosis revealed evidence of distant metastases. Furthermore, the first line treatment had to be surgery with curative intent, the pathological staging (tumour size and axillary lymph node status according to the pTpN classification) had to be known and tumour marker values of CEA and CA 15-3 had to be available at least within 30 days preceding surgery. A total of 1046 patients fulfilled these criteria. Also, histological grading, age and hormone receptor status were evaluated at the time of diagnosis.

Patients were treated with either modified radical mastectomy or lumpectomy and axillary node dissection with local radiotherapy and adjuvant systemic therapy if indicated, i.e. chemotherapy in node positive patients and hormone therapy in receptor positive patients. Regarding the course of disease, follow-up controls were performed with patient history, physical examination and laboratory tests including serum tumour markers CEA and CA 15-3, abdominal ultrasound, chest radiography, mammography and bone scan for detection of local or distant relapse. Additionally, computed tomography, MRI-tomography and other radiographs were carried out if necessary.

Patients with secondary contralateral breast cancer were excluded with respect to disease free survival. In 844 cases data regarding relapse were available. The median follow-up time for patients free of relapse at the time of analysis was 2.4 (range 0.2–12.7) years, and for those with relapse 1.8 (0.2–10.4) years. In 201 out of 844 patients recurrence of cancer occurred (first relapse: local recurrence *n*=68, distant metastases *n*=127, both *n*=6).

Regarding death from disease, out of 1046 patients 74 were lost to follow-up and the cause of death remained unclear in 21 cases. Nine hundred and fifty-one cases could be followed up, the number of women years was 3434. Ten patients died of causes other than cancer, and 95 women died from breast cancer. The median follow-up period for patients still alive or deceased from causes other than breast cancer was 3.0 (0.2–12.7) years and 2.9 (0.7–11.1) years for those deceased from breast cancer. The final data set is summarised in Table 1

**Table 1:**
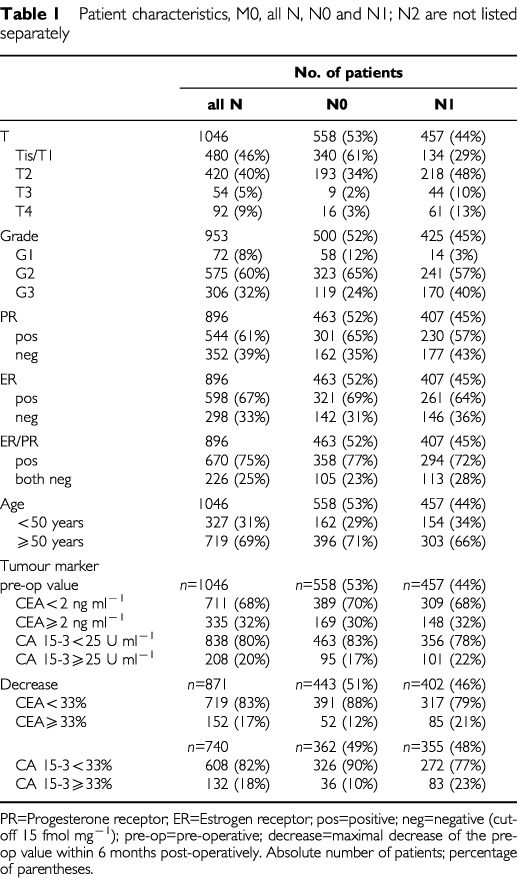
Patient characteristics, M0, all N, N0 and N1; N2 are not listed separately

.

### Marker analysis

The serum tumour markers CEA (IMx; MEIA, Abbott Laboratories, Chicago, IL, USA) and CA 15-3 (ES 700; Enzymun, Roche Diagnostics ((previously Boehringer Mannheim) Germany) were determined by automated test systems using sandwich ELISA assay kits. We investigated the values of the tumour markers CEA and CA 15-3 at the time of primary diagnosis. In addition to pre-operative values, tumour marker concentrations could be determined in a lower number of patients after primary therapy (see Table 1).

### Statistics

Patients were grouped according to tumour size (pTis and pT1/pT2/pT3/pT4), lymph node status, histological grading, age (<50/⩾50 years), hormone receptor status (cut-off 15 fmol mg^−1^), the pre-operative tumour marker level of CEA and CA 15-3 and the post-operative decrease of the tumour marker. For multivariate analyses patients with tumour size pT3 and pT4 were grouped together, as well as nodal status N1 and N2.

To define cut-off values of pre-operative tumour markers, we chose the 95%-percentile for healthy individuals of 2.0 ng ml^−1^ for CEA and 25 U ml^−1^ for CA 15-3 (Stieber, 1996). In addition to the pre-operative value we considered the difference of tumour-free or baseline values from initial pre-operative values. As individual baseline value we chose the first tumour marker value when primary therapy, including adjuvant therapy, was completed. For patients without adjuvant therapy the lowest value within 6 months after primary surgery was chosen. The difference of the pre-operative and the individual baseline value in per cent was called the post-operative decrease (cut-off: −33%, corresponding to the 90%-percentile).

Univariate survival curves for disease free survival and death from disease were estimated by the method of Kaplan–Meier (Kaplan and Meier, 1958) and differences between groups in survival or relapse-free time were tested using the log-rank test. Multivariate Cox regression analysis (Cox, 1972) was performed to identify those parameters having an independent significant influence on DFS and DFD and to calculate the hazard ratios.

All parameters found to be significant in univariate analyses were entered into multivariate Cox regression model and were excluded if their *P*-value was greater than 0.05. Interactions between all variables were investigated and the assumption of proportional hazards was tested by including time dependent variables in the model. All statistical analyses were carried out using SAS statistic software (SAS Institute, Inc., Cary, USA).

## RESULTS

**Figure 1:**
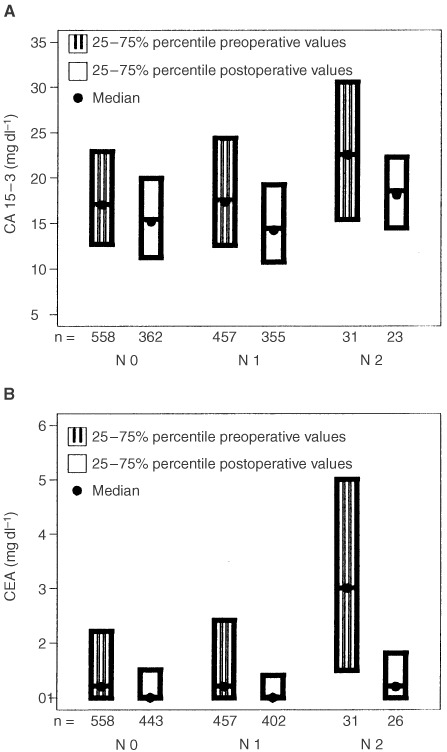
Values of CEA and CA 15-3 pre-operatively and post- operative baseline values.

Characteristics of the 1046 patients are listed in Table 1. The median pre-operative CEA value was 1.2 ng ml^−1^ (range: <1–47.8; 25%/75%-percentile: 1.0/2.3). The median pre-operative CA 15-3 value was 17.3 U ml^−1^ (range 3–1686; 25%/75%-percentile: 12.7/23.3). As shown in Figure 1

, only patients with N2 lymph node status had higher values. Post-operative tumour marker values were available in a smaller number of patients (CEA: *n*=871, CA 15-3: *n*=740). The median (range; 25/75% percentile) of the baseline values after surgery was 1.0 ng ml^−1^ (<1-15; 1.0/1.4) for CEA and 14.8 U ml^−1^ (3.5–120; 10.8/19.7) for CA 15-3 (Figure 1). For the post-operative decrease the respective values were 0% (−41-100; 0/23.1) for CEA and 12.2% (−43-94; 0/26.9) for CA 15-3. Tumour size, lymph node status, histological grading as well as hormone receptor status were found to be significant in univariate analysis. Age had no significant influence on disease free survival or death from disease. Elevated pre-operative values of CEA and CA 15-3 were associated with early death from disease (*P*=0.0001 for both markers), for relapse high levels of CA 15-3 were also significant (*P*=0.0003), whereas elevated values of CEA only showed borderline significance (*P*=0.064). Individual baseline values as defined above had no prognostic value with either disease free survival or death from disease, as end points.

A decrease of more than 33% of the CEA value from the pre-operative level was associated with early relapse and death from disease (both *P*=0.0001). A decrease of CA 15-3 of more than 33% was an indication of bad prognosis (death from disease *P*=0.007; disease free survival *P*=0.0087).

**Figure 2:**
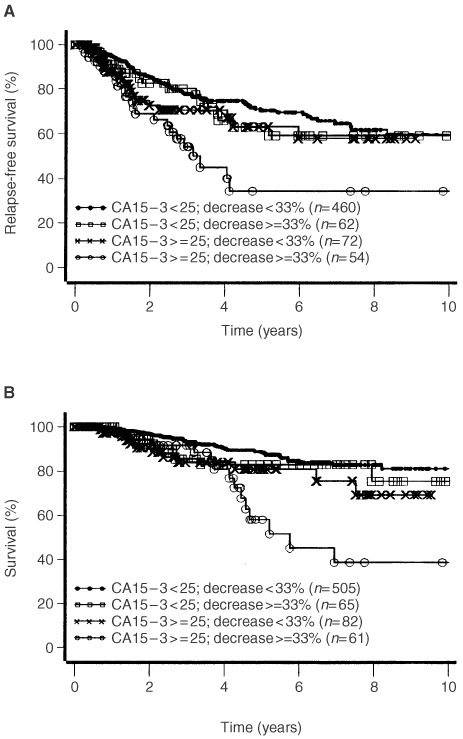
Disease free survival (**A**, log-rank test: *P*=0.0007), and survival until death of disease (**B**, log-rank test: *P*=0.0005) according to CA 15-3 pre-operative values and post-operative decrease.

Kaplan–Meier curves for DFS and DFD are shown for the combination of pre-operative value and post-operative decrease of CEA and CA 15-3, respectively (Figures 2

and 3

**Figure 3:**
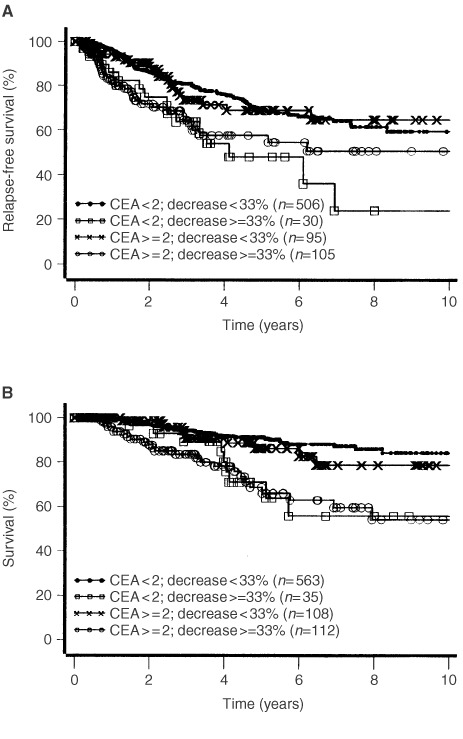
Disease free survival (**A**, log-rank test: *P*=0.0003) and survival until death of disease (**B**, log-rank test: *P*=0.0001) according to CEA pre-operative values and post-operative decrease.

). Those patients whose elevated pre-operative values of CA 15-3 decreased more than 33% had the highest risk of relapse and death from cancer (Figure 2). In contrast to CA 15-3, the CEA decrease alone, independent from the level of the pre-operative value, was a strong predictor for early relapse and death of breast cancer (Figure 3).

In multivariate analysis, independent prognostic factors for DFS in 658 patients with 160 recurrences were tumour size, lymph node status, hormone receptors and decrease of the pre-operative CEA value ⩽33% (Table 2

**Table 2:**
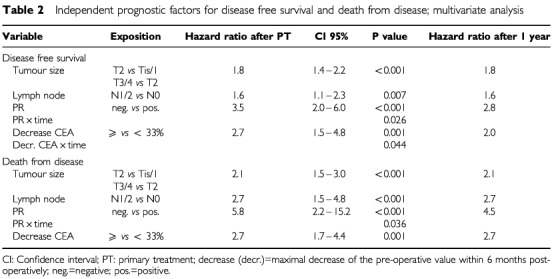
Independent prognostic factors for disease free survival and death from disease; multivariate analysis

). It is notable that 1 year after primary therapy the hazard ratio for relapse for both decrease of CEA values and progesterone receptor diminished compared with the initial risk. Tumour grade, CA 15-3 and pre-operative values of CEA were without significance for relapse.

In 720 patients with 74 tumour associated deaths, tumour size, lymph node status, hormone receptor status and the decrease of CEA were independent predictors for death from disease in multivariate analysis (Table 2). In contrast to relapse-free survival, only the hazard ratio for the hormone receptor status was influenced by time, the decrease of CEA level showed no time dependency. Likewise as for DFS, grading, CA 15-3 and the pre-operative values of CEA were without influence on death from disease.

## DISCUSSION

CEA and CA 15-3 are the most thoroughly investigated serum tumour markers in breast cancer. It is generally agreed that tumour markers in breast cancer patients are not a tool for primary diagnosis, because of their low sensitivity and specificity (Tondini *et al*, 1989; Fateh-Moghadam and Stieber, 1993; Lamerz *et al*, 1993). Their use for early detection of metastases seems to be promising (Lamerz *et al*, 1991; Stieber *et al*, 1992; Vizcarra *et al*, 1994; O'Hanlon *et al*, 1995) and their use for measuring therapeutic response in metastatic disease is widely accepted (Tondini *et al*, 1988; Dnistrian *et al*, 1991; Robertson *et al*, 1991; Safi *et al*, 1991). Many studies tried to assess the prognostic role of these tumour markers (some analysed in serum, some in tissue), but most of them had low patient numbers or short follow-up periods, and used only univariate analyses (Myers *et al*, 1978; Tormey and Waalkes, 1978; Bezwoda *et al*, 1981; Mansour *et al*, 1983; Kallioniemi *et al*, 1988; Hammer *et al*, 1992; O'Hanlon *et al*, 1995). To our knowledge, there is no multivariate analysis on CEA in serum, only a few studies performed multivariate analyses on CEA in breast cancer tissue (Esteban *et al*, 1994; Sundblad *et al*, 1996). Among the two multivariate analyses of CA 15-3 in serum at the time of diagnosis of breast cancer (Gion *et al*, 1991; Shering *et al*, 1998), only Shering had a sufficiently high number of patients. To date, no study has tested both tumour markers CEA and CA 15-3 for independent prognostic value at the time of primary intervention in breast cancer patients.

In the present study, we investigated the association of the serum levels of the tumour markers CEA and CA 15-3 with DFS and DFD in women with breast cancer without metastases at the time of primary diagnosis, in relation to age and the established prognostic factors tumour size, lymph node status, histological grading and hormone receptor status. In accordance to other studies, we found tumour size, lymph nodes, histological grading and hormone receptors to be prognostically significant for DFS and DFD (Chevallier *et al*, 1988; Carter *et al*, 1989; Nomura *et al*, 1992; Carriaga and Henson, 1995).

In most patients, tumour marker levels were found to be very low (Figures 1 and 2), but were higher than average levels in healthy women (CEA 1.0 ng ml^−1^; CA 15-3 13.6 U ml^−1^) (Stieber, 1996). We defined the marker cut-off values in our study by the 95 %-percentile of healthy individuals (2.0 ng ml^−1^ for CEA and 25 U ml^−1^ for CA 15-3).

We found that elevated pre-operative serum marker values were correlated with early relapse (CA 15-3; *P*=0.0003) and death from disease (CEA, CA 15-3; *P*=0.0001 both) in univariate analyses. Possibly, the release of tumour associated antigens at the time of diagnosis proves blood supply respectively vascularisation of the tumour and by consequence the possibility of already existing micrometastases and bad prognosis from the beginning (Gasparini *et al*, 1997).

By comparing pre- and post-operative values we found a decline in values post-surgery. The post-operative marker values we determined were well comparable to the median of healthy individuals which could be expected as we investigated only patients with complete resection of the tumour (R0 resection). Therefore it is understandable that we found no significant correlation with the recurrence of disease assessing isolated post-operative marker values of CEA and CA 15-3. Other studies that found an association between elevated post-operative levels of CEA and recurrence of disease had only small patient numbers, short follow up and partly investigated advanced stages of disease (Myers *et al*, 1978; Hammer *et al*, 1992). This is one of the first studies to relate changes in serum marker levels following surgery to patient outcome.

In those patients where marker levels of CEA decreased more than 33%, a significantly higher risk for relapse and death from disease (both *P*=0.0001) in univariate analyses was observed. The general opinion that R0-resection corresponds to good prognosis is not in contradiction to our findings, because we only observed patients with complete resection of the tumour.

It is surprising that we found the high prognostic relevance of the decrease only for CEA, whereas for CA 15-3 a significant worse prognosis can be seen only for patients with high pre-operative tumour marker values and significant decrease after surgery (Figures 2 and 3). This could probably be due to the fact that up to now the reproducibility of CA 15-3 values at low concentrations is unsatisfactory.

In multivariate analysis, this decrease of CEA proved to be an independent prognostic factor (Table 2). The results for CA 15-3 were comparable to CEA in univariate analyses but showed no significance in multivariate analysis at least when both markers were included in the model simultaneously. This could be due to the fact that the correlation with tumour size is higher for CA 15-3 than for CEA.

As we determined both markers, CEA and CA 15-3, it is difficult to compare our study with the two other relevant studies of Gion and Shering (Gion *et al*, 1991; Shering *et al*, 1998). Although Gion found, in accordance to us, that pre-operative serum levels of CA 15-3 had no prognostic relevance in multivariate analysis, the number of patients and the unknown number of relapse and death from disease is probably too small to give reliable results.

In contrast Shering *et al* (1998) reported on a high number of patients and found a prognostic relevance for pre-operative CA 15-3 values. The prognostic relevance of a single factor in multivariate analysis depends on which and how other factors are included in the model. Thus, our results would be similar to those of Shering *et al* (1998), if CEA was not included in the analysis.

### Conclusion

In this study the post-operative decrease of the serum tumour marker CEA was a strong independent prognostic factor for disease free survival and death from disease in breast cancer patients. To our knowledge, this is the first study reporting on the high prognostic relevance of the decline of tumour associated antigens. It is also the largest study to-date to have analysed the prognostic value of serum tumour markers in breast cancer. An advantage of our approach for clinical practice would be the independence from tumour tissue.

In addition to those prognostic factors being already indicative for adjuvant therapy like lymph nodes and grading, a randomized prospective therapy intervention study based on the decrease of CEA would be needed to prove the relevance of our findings.
